# Protein Kinase R-like Endoplasmic Reticulum Kinase-Mediated ER-Mitochondria Coupling Regulates Odontogenic Differentiation of Human Dental Pulp Stem Cells Under Inflammatory Stimuli

**DOI:** 10.1016/j.identj.2026.109440

**Published:** 2026-03-05

**Authors:** Yiqing Wang, Yiqiao Li, Yu Jin, Zhipu Luo, Ruirui Liu

**Affiliations:** a College of Stomatology, Key Laboratory of Shaanxi Province for Craniofacial Precision Medicine Research, Xi'an Jiaotong University, Xi'an, China; b Shanghai Pudong New Area Gongli Hospital, Shanghai, China

**Keywords:** Dental pulp, Stem cells, Endoplasmic reticulum stress, Mitochondria-associated membranes

## Abstract

**Introduction and aims:**

Human dental pulp stem cells (hDPSCs) play pivotal roles in the regeneration of pulp-dentin complex, yet their odontogenic differentiation is critically modulated by the inflammatory microenvironment. Protein kinase R-like endoplasmic reticulum kinase (PERK), a key regulator of endoplasmic reticulum stress, is highly enriched in mitochondria-associated endoplasmic reticulum membranes (MAMs) and exerts critical functions. However, its precise mechanisms in inflammatory regulation and cellular differentiation remain elusive. This study elucidates the PERK-centred regulatory mechanism in MAMs that governs inflammation-impaired odontogenic differentiation of hDPSCs, potentially involving IP3R-dependent calcium flux and dynamic protein interactions in MAMs.

**Methods:**

Rat pulpitis models and in vitro lipopolysaccharide (LPS)-induced inflammatory models of hDPSCs were established to investigate the effects of PERK signalling in odontogenesis under inflammatory conditions. Lentivirus-mediated silencing of PERK was performed to evaluate its role in LPS-induced inflammation. Molecular mechanisms were analysed using RNA sequencing, immunofluorescence, and transmission electron microscopy analyses.

**Results:**

LPS stimulation activated the PERK signalling pathway, significantly upregulating MAM-related molecules (IP3R, VDAC1, GRP75) and enhancing PERK/VDAC1 colocalization and the formation of endoplasmic reticulum-mitochondria coupling structures. PERK silencing effectively mitigated LPS-induced mitochondrial swelling, ER dilatation, and calcium influx dysregulation, while restoring alkaline phosphatase activity and odontogenic differentiation potential. Mechanistically, PERK suppressed hDPSC mineralization by modulating IP3R-mediated calcium signalling pathway in MAMs.

**Conclusion:**

This study demonstrates that LPS-induced inflammatory stress reprograms hDPSCs bioactivity via PERK-centric control of MAMs likely through quantitative enhancement, structure specialization, and functional potentiation. The underlying mechanisms may involve IP3R-mediated regulation of calcium ion influx and protein interactions within MAMs.

## Introduction

Dental pulp-dentin complex (PDC) damage, caused by caries, trauma, or iatrogenic interventions, is commonly accompanied by inflammatory cascades. Human dental pulp stem cells (hDPSCs), as pluripotent precursor cells, exhibit remarkable plasticity by dynamically balancing proliferation and differentiation in response to microenvironmental cues via immunomodulation, resolution of inflammation, and deposition of mineralized matrices mechanisms.^1^ The reparative outcome of PDC damage hinges on the equilibrium between extrinsic stress magnitude and the intrinsic self-repair capacity of hDPSCs.^2^ The nature and intensity of external stimuli directly impact the homeostasis between hDPSCs differentiation and apoptosis. Typically, PDC maintains a baseline level of defence against external stimuli. Under mild to moderate stimuli, hDPSCs initiate self-repair mechanisms, differentiate into odontoblast-like cells, and secrete extracellular matrix components such as fibronectin and type III collagen to repair PDC. However, excessive stimuli overwhelm hDPSCs tolerance threshold, triggering apoptotic cascades via apoptotic pathways, including mitochondrial apoptosis, death receptor pathways, endoplasmic reticulum stress (ERS), p53 signalling, and MAPK signalling.^3^^,^^4^ Strategic modulation of the intensity and duration of stimuli, along with bolstering hDPSCs stress adaptability, holds significant clinical value for pulp diseases management. Exploring the molecular mechanisms and regulatory pathways underlying hDPSCs responses to external stimuli may provide new strategies and theoretical insights for pulp disease prevention and regenerative endodontics.

Protein kinase R-like endoplasmic reticulum kinase (PERK), a transmembrane protein located on the ER membrane, serves as a pivotal mediator of the unfolded protein response (UPR). PERK critically regulates various biological processes, including inflammation, cell proliferation, and differentiation. Mechanistically, lipopolysaccharide (LPS)-induced inflammatory stimuli trigger ERS, thereby activating the PERK signalling axis, which amplifies inflammatory responses and apoptotic pathways.^5^ Inhibition of the PERK pathway can attenuate ERS-related inflammation, highlighting its potential role in stress adaptation.^6^ Paradoxically, context-specific PERK activation may exert anti-inflammatory effects. For instance, activating the PERK/Nrf2 pathway with berberine in acute lung injury models suppressed IL-2 and IL-1 expression, mitigating LPS-induced inflammation.^7^ These discrepancies likely attributed to cell type-specific signalling dynamics, variations in stimulus intensity, and temporal activation patterns, underscoring the need for nuanced interpretation of PERK’s role in inflammation. PERK has emerged as a key regulator of mineralized tissue homeostasis. Under physiological stimuli, PERK activation via ATF4 promotes osteogenesis. However, under pathological stimuli, hyperactivation of PERK triggers apoptotic pathways, leading to osteoblast apoptosis, disrupting bone metabolism, and inhibiting osteogenesis. Our previous work revealed that PERK signalling was activated in an LPS-induced inflammatory microenvironment, suppressing hDPSCs odontogenic differentiation capacity, a phenotype rescued by PERK knockdown. However, the precise molecular mechanisms linking PERK-mediated signalling to hDPSCs fate determination remain unresolved, warranting further investigation into its dualistic regulatory roles.

Mitochondria-associated endoplasmic reticulum membranes (MAMs) are tight yet nonfusional contact regions between the mitochondrial outer membrane and the ER membrane, mediated by specific proteins.^8^ Recent studies have identified PERK enrichment in MAMs, where its functional integrity is essential for mitochondrial protein quality control.^9^ By engaging with MAMs-resident protein complexes, PERK orchestrates mitochondrial ultrastructure remodelling, proteostatic balance, and metabolic reprogramming. Genetic ablation of PERK disrupts ER architecture, diminishes MAMs abundance, and dysregulates calcium flux between ER and mitochondria – a critical signalling axis for cellular homeostasis.^10^ Strikingly, PERK mutants lacking kinase activity can restore MAMs connectivity, suggesting that PERK regulates MAMs formation and function independently of UPR.^11^ However, the precise mechanisms intermediates and spatiotemporal dynamics of PERK-MAMs crosstalk still requires further exploration. Whether PERK-mediated MAMs remodelling in modulating hDPSCs odontogenic differentiation under inflammatory microenvironments is still unclear.

This study aims to elucidate the role of PERK in regulating ER-mitochondria coupling and MAM-mediated hDPSCs odontogenic differentiation under inflammatory conditions. The findings may provide a molecular basis for PDC repair mechanisms and novel strategies for vital pulp preservation and related disease prevention.

## Materials and methods

### Cell isolation, culture, and treatment

This study was approved by the Ethics Committee of Xi’an Jiaotong University Dental Hospital, and written informed consents were obtained from all participants. Dental pulp tissues were collected from healthy third molars or premolars extracted from individuals aged 18 to 25 years for orthodontic or impaction-related reasons. Inclusion criteria required that the teeth were free of caries, periodontal diseases, and periapical lesions.

Following extraction, teeth were immediately stored in α-MEM medium supplemented with 10% antibiotics (penicillin-streptomycin). Under sterile conditions, pulp tissues were isolated, minced, and digested with 3 mg/mL type I collagenase. After centrifugation, the pellet was resuspended in complete α-MEM containing 20% foetal bovine serum and cultured at 37°C under 5% CO_2_. Primary hDPSCs (P0) were passaged using 0.25% trypsin-EDTA upon reaching 80% to 90% confluence, and cells from passages 2 to 5 were utilized for experiments. For inflammation modelling, hDPSCs were treated with LPS (Sigma) at 0, 10, and 20 µg/mL for specified durations.

### RNA sequencing and bioinformatics analysis

Cells were divided into three groups: Control, LPS, and ShPERK, with three independent biological replicates per group. Lentiviral transduction was performed using a complete antibiotic-free medium with MOI of 20 and Polybrene at 5 μg/mL. The Control and LPS groups received virus-free antibiotic-free medium, while the ShPERK group was transduced with the PERK-targeting shRNA lentivirus in antibiotic-free medium. Total RNA was extracted following 6 hours of stimulation with 10 μg/mL LPS working solution, and the purity and concentration were measured. High-quality RNA samples were selected for subsequent library construction. RNA quality assessment is detailed in Supplementary Table 1. Sequencing libraries quality was assessed using the Agilent 2100 Bioanalyzer system (Agilent Technologies).

Sequencing libraries were pooled and sequenced on a high-throughput platform. Libraries were pooled and sequenced on an Illumina NovaSeq 6000 platform in paired-end (150 bp) mode. The sequencing yielded an average of approximately 34.5 million read pairs per sample, ensuring sufficient depth for subsequent analysis. The raw sequencing depth and quality metrics (Q30) for each sample are detailed in Supplementary Table 2. Raw data underwent quality control, preprocessing, and statistical analysis. Differentially expressed genes (DEGs) were identified with a threshold of |log2FoldChange| > 1 and *P* value <.05. Kyoto Encyclopedia of Genes, Genomes (KEGG) pathway enrichment and gene ontology analyses were performed. Visualizations, including scatter plots, volcano plots, and heatmaps, were generated.

### Animal experiments

Twenty-four specific pathogen-free male SD rats (7-8 weeks old, 180-220 g) with normal, healthy dentition were selected. A total of 24 rats were randomly allocated into two main treatment groups: the dentin defect group (*n* = 12) and the pulp exposure group (*n* = 12). Within each group, animals were further randomly assigned to four time points (days 0, 1, 7, and 14), resulting in *n* = 3 rats per subgroup per time point. Animals were placed under general anaesthesia with isoflurane (2% in 100% O_2_) and maintained throughout the entire surgical procedure. Inflammation models were established in the upper first molars using a high-speed dental drill with G1 round burs. The rats were randomly allocated into two groups based on the treatment applied: pulp exposure group (pulp was penetrated with sterile probes) and dentin defect group (Cavities approximately 1 mm deep were prepared without pulpal exposure).

Animals were euthanized on days 0, 1, 7, and 14 postoperation. Maxillae containing the first molars were collected, fixed in 4% paraformaldehyde for 48 hours, washed overnight, and decalcified in 10% EDTA. Samples were dehydrated with ethanol, embedded in paraffin, and sectioned (7 μm thickness) using a Leica CM1950 microtome (Leica). Sections were stored at room temperature for further analysis.

### Quantitative real-time PCR (qRT-PCR)

Total RNA was extracted using TRIzol reagent, followed by reverse transcription using the Evo M-MLV kit (Accurate Biology). Expression levels were measured via SYBR Green Pro Taq HS master mix (Accurate Biology). GAPDH served as the reference gene, and the primer sequences are delineated in Table.

**Table:** Primer sequences used for qRT-PCR.

Gene	Sequences (5′-3′)
H-GAPDH-F	TGTGTCCGTCGTGGATCTGA
H-GAPDH-R	TTGCTGTTGAAGTCGCAGGAG
H-IL-1-F	CCAGGGACAGGATATGGAGCA
H-IL-1-R	TTCAACACGCAGGACAGGTACAG
H-TNF-α-F	CTGCCTGCTGCACTTTGGAG
H-TNF-α-R	ACATGGGCTACAGGCTTGTCACT
H-IL-6-F	TCCAGTTGCCTTCTTGGGAC
H-IL-1-R	GTACTCCAGAAGACCAGAGG
H-PERK-F	AAGCACCACCAGAGAAGTGG
H-PERK-R	GTGCATCCATTGGGCTAGGA
H-IP3R-F	GCGGAGGGATCGACAAATGG
H-IP3R-R	TGGGACATAGCTTAAAGAGGCA
H-VDAC1-F	ACGTATGCCGATCTTGGCAAA
H-VDAC1-R	TCAGGCCGTACTCAGTCCATC
H-GRP75-F	TGCATCAGAAGCAATCAAGG
H-GRP75-R	TGGCCCAAGTAATTTTCTGC

### Western blot

The total protein concentration of hDPSCs samples from each group was measured using a BCA protein assay kit (Beyotime). Proteins were separated using 8% SDS-PAGE gel electrophoresis at 110 V for 1.5 hours and transferred onto PVDF membranes (Millipore). The membranes were incubated overnight at 4°C with primary antibodies: anti-PERK antibody (1:5000, Wuhan Sanying, rabbit origin) and anti-β-actin antibody (1:500, Santa Cruz Biotechnology, mouse origin). After washing, membranes were incubated for 1 hour at room temperature with secondary HRP-conjugated antibodies: goat anti-rabbit IgG (1:5000, Bioss) and goat anti-mouse IgG (1:5000, Bioss). Protein band was visualized by enhanced chemiluminescence reagent (NCM Biotech), and the intensities were quantified using ImageJ software.

### Immunofluorescence

Paraffin sections were dewaxed in xylene and rehydrated through an ethanol gradient. Antigen retrieval was performed, and sections were blocked with 10% goat serum (BSA) for 1 hour at room temperature. Primary antibodies, including anti-PERK (1:300, rabbit), anti-VDAC1 (1:100, mouse), and anti-IP3R (1:100, rabbit), were applied overnight at 4°C. After washing, sections were incubated with secondary antibodies at room temperature for 1 hour. Sections were mounted with DAPI-containing antifade solution and visualized under a laser confocal microscope (Olympus FV3000).

### Lentiviral PERK silencing and efficiency detection

PERK-silencing lentiviral vectors were purchased from Gene Pharma. Based on a preliminary titration experiment, MOI = 20 was determined to be optimal for achieving high transduction efficiency with minimal cytotoxicity and was used in all subsequent silencing experiments. Cells were divided into three groups: control, ShPERK, and ShPERK-NC. After 48 hours of transduction, the medium was replaced, and cells were cultured for an additional 72 to 96 hours. The transduction efficiency was evaluated using a fluorescent inverted microscope.

### Mitochondrial Ca^2+^ concentration detected by Rhod-2 AM fluorescent probe

Cells were harvested and washed gently with prewarmed HBSS containing calcium and magnesium. Rhod-2 AM working solution (Yeasen Biotech) was applied to indicate the Ca^2+^ concentrations in cells, with cell incubated at 37°C in the dark for 30 minutes to ensure complete de-esterification. Fluorescence labelling and cell morphology were observed using an inverted fluorescence microscope (Zeiss), and images were analysed.

### Transmission electron microscopy observation of ER and mitochondrial morphology, and structural changes of MAMs

Cells from each group were fixed with glutaraldehyde in the dark at room temperature for 30 minutes, followed by fixation with osmium tetroxide for over 2 hours. Samples were dehydrated through an ethanol gradient, embedded in epoxy resin, sectioned, and stained with uranyl acetate and lead citrate. Images were captured using a transmission electron microscope (Hitachi), and mitochondrial and ER ultrastructural features were analysed using ImageJ software.

### Alkaline phosphatase (ALP) staining

Cells were fixed with 4% paraformaldehyde and stained with BCIP/NBT solution. The staining reaction was performed at room temperature in the dark for 30 minutes. Then cells were gently rinsed with distilled water to remove excess dye. Mineralized nodule formation was visualized under a light microscope and captured for further analysis.

### Statistical analysis

Results were expressed as mean ± standard deviation and analysed using GraphPad Prism 9.0 software. Student’s *t* test or one-way ANOVA was used for comparisons. Correlations were assessed using Pearson’s correlation coefficient. *P < .05; **P < .01; ***P < .001; ****P < .0001; ns, not statistically significant.

## Results

### Bioinformatics analysis of the mechanism of PERK regulating hDPSC functional activity under LPS-induced inflammation

**Fig. 1:**
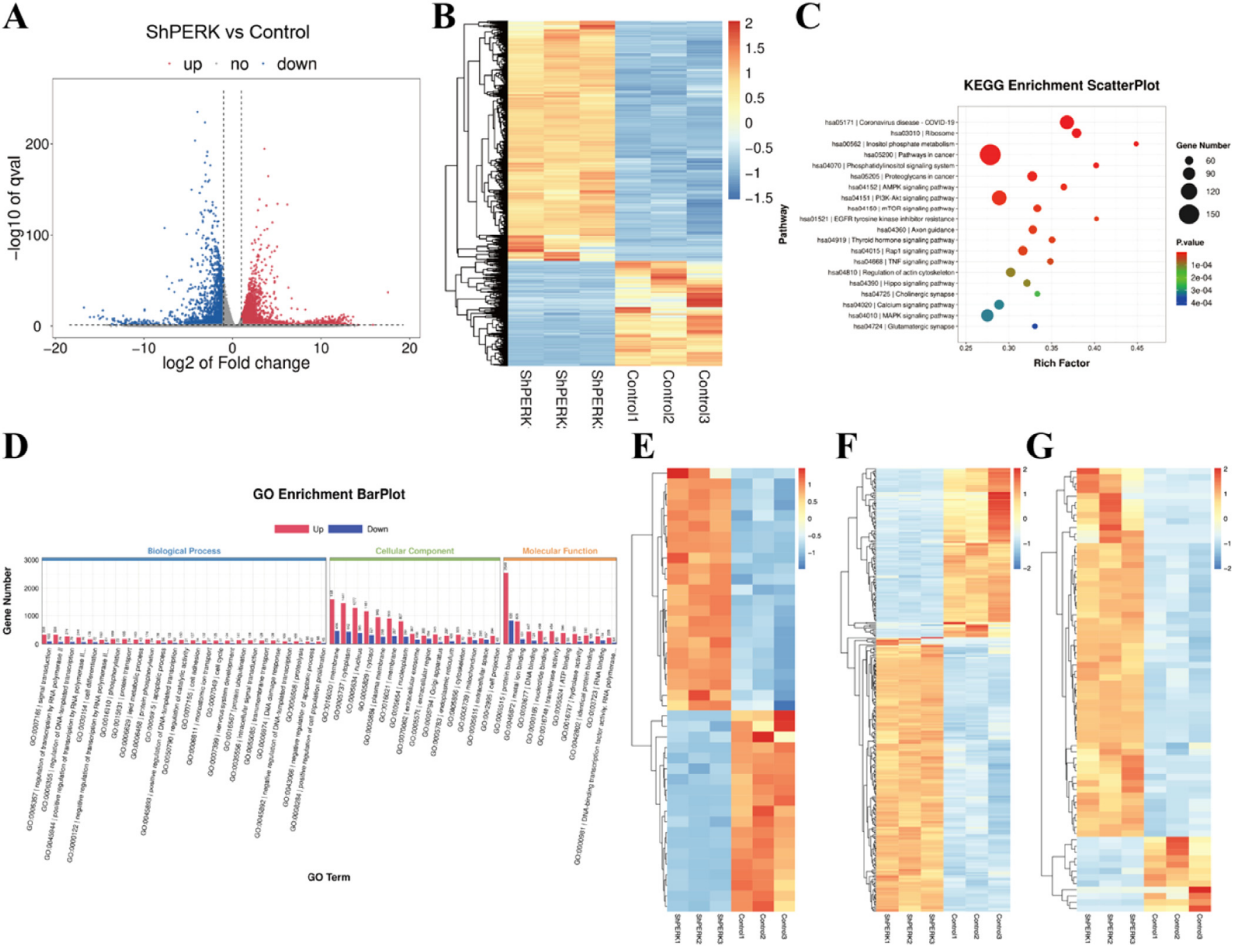
Transcriptomic profiling and mechanistic insights into PERK-mediated regulation of hDPSCs. (A) Volcano plot of differentially expressed genes (DEGs) between ShPERK and control groups (blue: 4176 upregulated genes; red: 1827 downregulated genes; |log2FoldChange| > 1 and *P* value <.05). (B) Hierarchical clustering heatmap of DEGs between ShPERK and control groups. (C and D) KEGG enrichment bubble plot and GO enrichment analysis of DEGs in ShPERK vs control groups. (E-G) Pathway-centric hierarchical clustering heatmaps of DEGs associated with specific biological processes: (E) Endoplasmic reticulum stress (ERS)-related genes (42 genes). (F) Mitochondrial function-related genes (315 genes). (G) Calcium signalling-related genes (71 genes).

Bioinformatics analysis confirmed that LPS stimulation triggered a robust inflammatory transcriptomic response in hDPSCs, with significant upregulation of key inflammatory pathways, including TNF, IL-17, and NOD-like receptor signalling pathways (Figure S1A-C). In the ShPERK group, 4176 genes were upregulated, and 1827 were downregulated compared to the control group (Figure 1A,B). KEGG enrichment analysis of these DEGs indicated PERK silencing significantly affected cellular signalling and homeostasis pathways such as calcium, MAPK, and PI3K-Akt signalling pathways (Figure 1C). The enrichment of calcium signalling pathway implies PERK regulates intracellular calcium dynamics, and MAPK pathway enrichment suggests a potential regulatory role for MAMs integrity. Gene ontology analysis further delineated the multifaceted impact of PERK loss (Figure 1D). The significantly perturbed biological processes encompassed protein phosphorylation, ubiquitination, lipid metabolism, signal transduction, and cell differentiation, reflecting broad disruption in protein processing and cellular signalling – functions intimately linked to ER and MAMs activity. At the cellular component level, membrane, endoplasmic reticulum, and mitochondrion were most affected, aligning with PERK’s subcellular localization. These findings collectively highlight PERK as a critical modulator of MAMs-related functions, influencing calcium flux, interorganelle communication, and downstream signalling cascades.

Hierarchical clustering analysis on DEGs elucidate the downstream transcriptional cascades. The heatmaps demonstrated clear segregation between the ShPERK and control groups, revealing distinct, pathway-coherent expression signatures. Analysis of the 42 ERS-related DEGs showed a bifurcated expression pattern involving the induction of proapoptotic transcription factors such as ATF4 and DDIT3 (CHOP), coupled with altered expression of ER chaperones including PDIA3 and PDIA4 (Figure 1E). Concordantly, 315 mitochondrial function-related genes exhibited a broad downregulatory trend. Key subunits of the oxidative phosphorylation system, such as COX5B and NDUFA13, were suppressed, indicating compromised mitochondrial bioenergetics (Figure 1F). Furthermore, we observed a pronounced upregulation in 71 calcium signalling-related genes, notably the ER calcium release channel IP3R3 (gene symbol: ITPR3) and the voltage-gated calcium channel CACNA1D (Figure 1G). Collectively, these transcriptomic profiles illustrate that PERK silencing mitigates the LPS-induced disruption by concurrently attenuating sustained ERS, restoring mitochondrial integrity, and normalizing calcium signalling regulators, including IP3R.

### PERK activation and colocalization with VDAC1 in MAMs in a rat pulp-dentin injury model

**Fig. 2:**
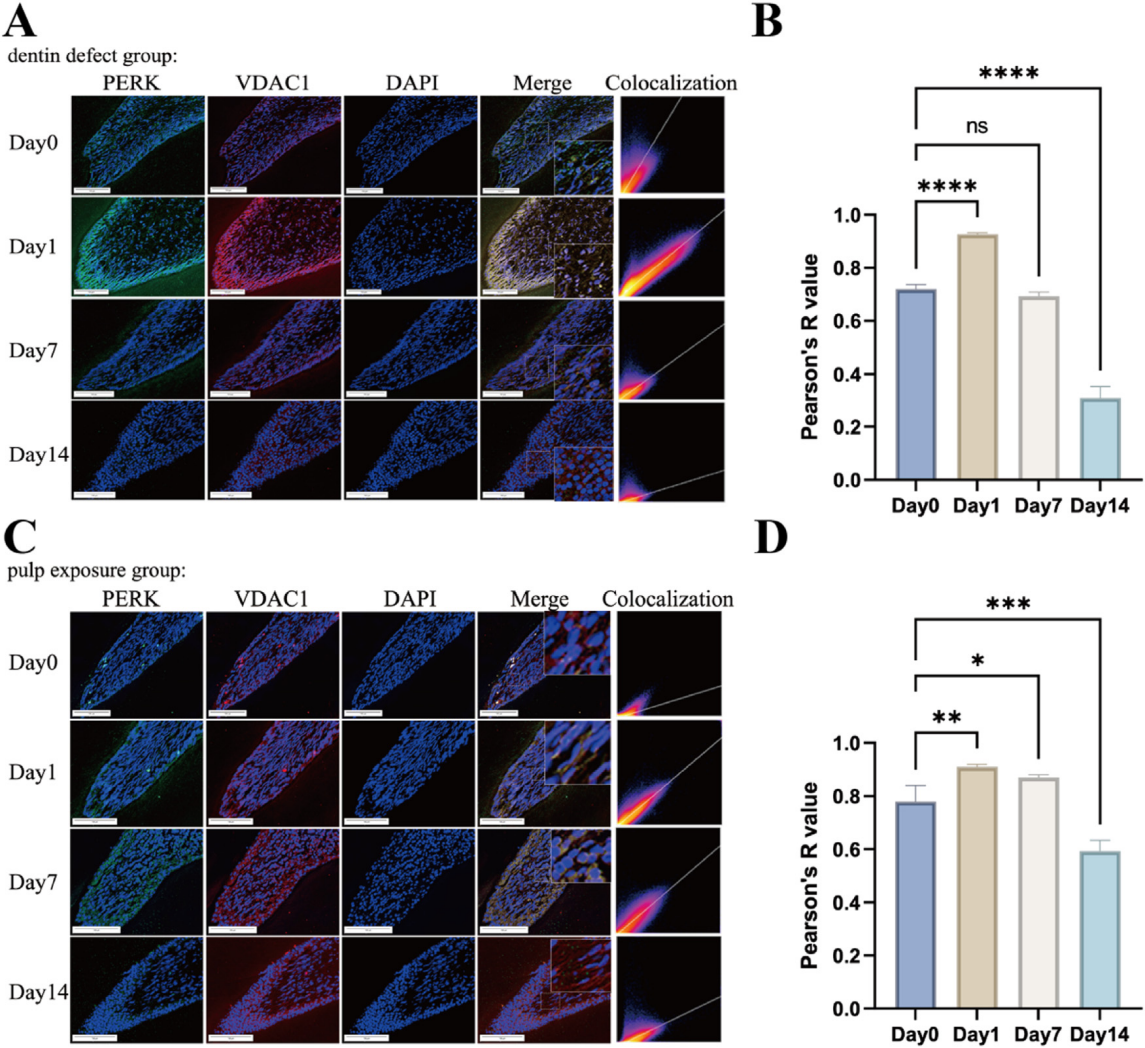
PERK-mediated endoplasmic reticulum-mitochondria coupling in inflammatory animal models. (A) Immunofluorescence double staining of PERK (red) and VDAC1 (green) in the dentin defect group at day 0, day 1, day 7, and day 14 postinjury. Colocalization analysis (yellow) is shown in merged images; scale bar = 100 μm. (B) Quantification of PERK-VDAC1 colocalization in the dentin defect group using Pearson’s correlation coefficient (*r*). Data expressed as mean ± SEM (*n* = 5/group). (C) Immunofluorescence double staining of PERK and VDAC1 in the pulp exposure group at day 0, day 1, day 7, and day 14 postexposure. Colocalization signals (yellow) are indicated; scale bar = 100 μm. (D) Statistical analysis of PERK-VDAC1 colocalization in the pulp exposure group.

Immunofluorescence double staining analysis of the dentin defect group revealed that PERK and VDAC1 expression levels significantly increased on day 1, and colocalization analysis quantified by Pearson’s correlation coefficient demonstrated a significant importance (Figure 2A). The colocalization relationship did not change significantly on day 7 but decreased on day 14, indicating an initial rise followed by a decline (Figure 2B). In the pulp exposure group, the results (Figure 2C) showed significant elevations in PERK and VDAC1 expression levels on days 1, 7, and 14 and a significant colocalization expression in PERK and VDAC1 on days 1 and 7 (Figure 2D).

### PERK modulates MAMs integrity in hDPSCs under LPS-induced inflammation

**Fig. 3:**
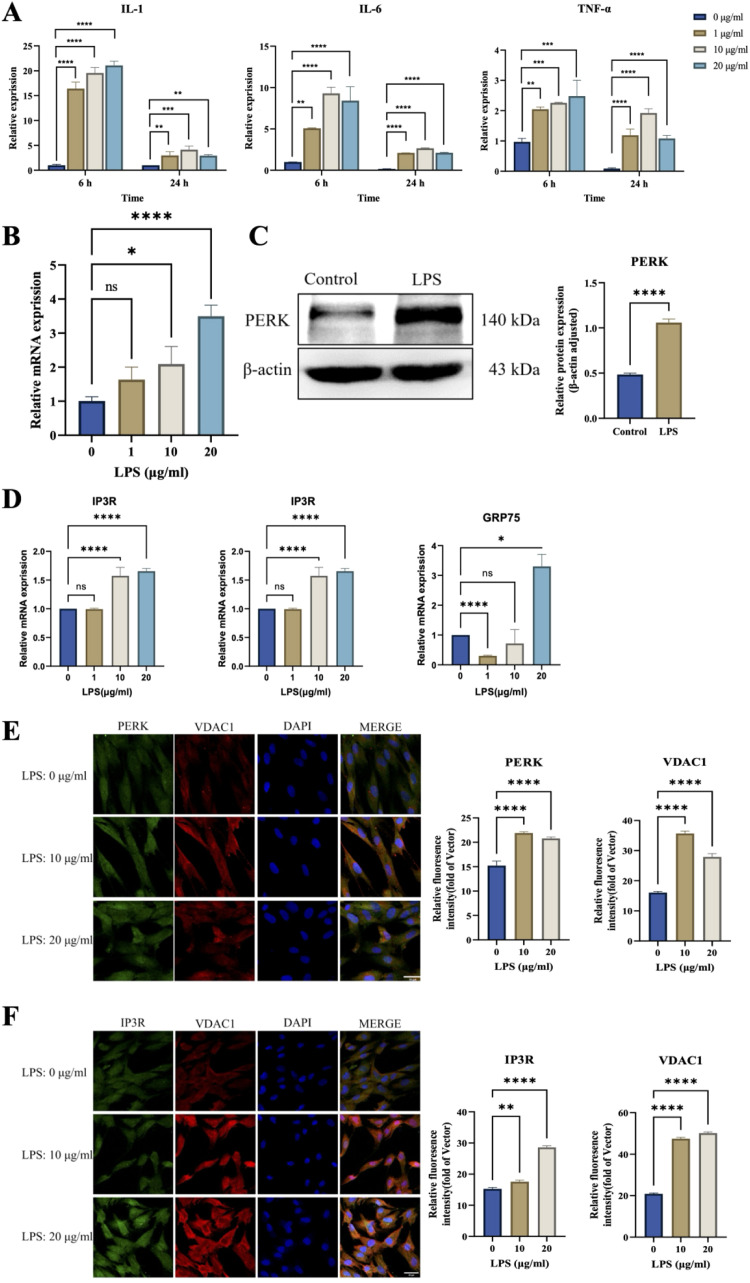

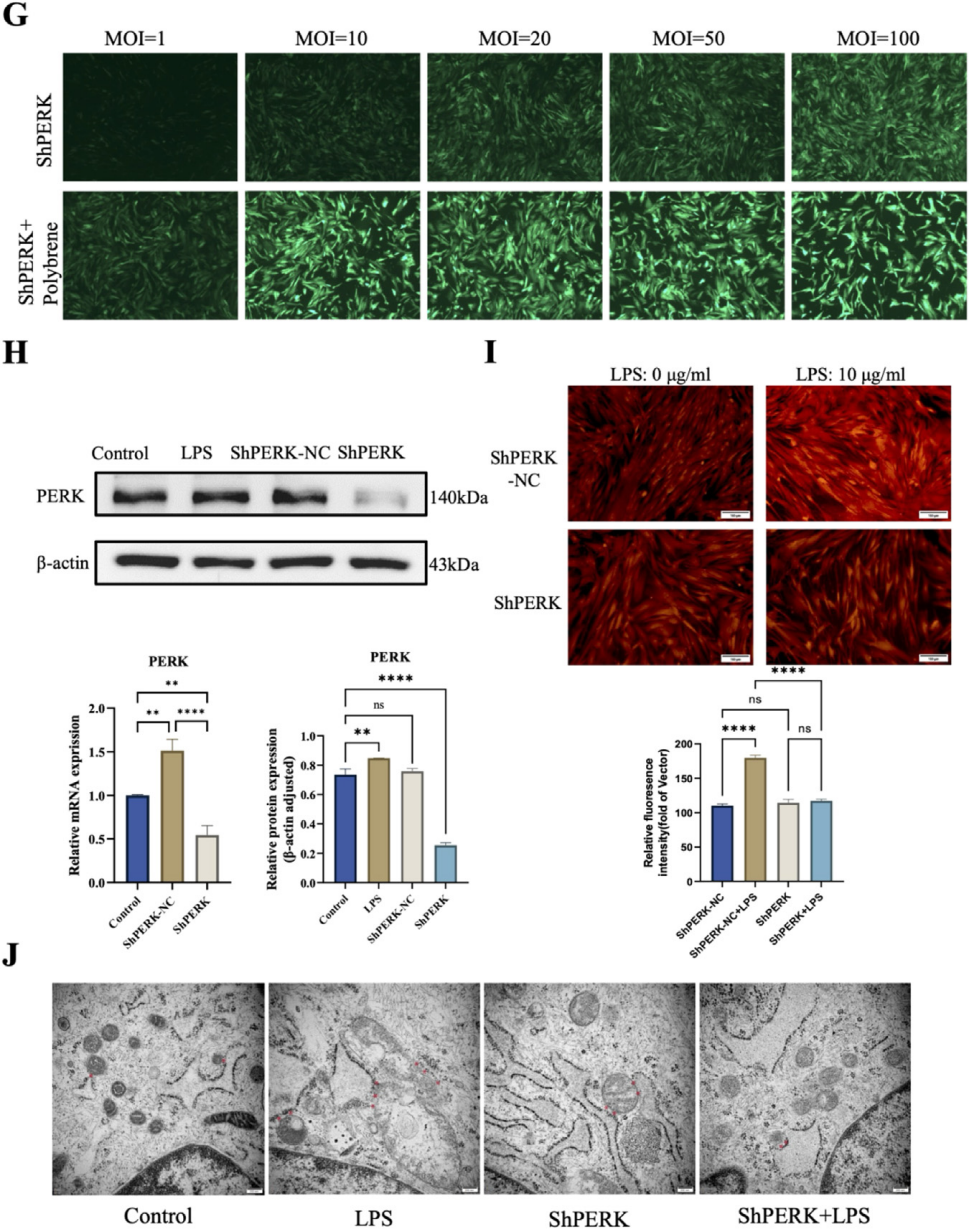
Inflammation-induced PERK-dependent MAMs remodelling in hDPSCs. (A) Expression levels of inflammatory cytokines (IL-1β, IL-6, TNF-α) in hDPSCs treated with LPS gradient concentrations (0, 1, 5, 10 μg/mL) for 6 and 24 hours. (B) PERK mRNA expression in hDPSCs after 6 hours LPS stimulation. (C) Western blot analysis of PERK protein expression in hDPSCs treated with 10 μg/mL LPS for 6 hours. (D) mRNA levels of MAMs-associated proteins (IP3R, VDAC1, GRP75) in LPS-stimulated hDPSCs. (E) Immunofluorescence costaining of PERK (red) and VDAC1 (green) in LPS-treated hDPSCs. Quantitative analysis of PERK and VDAC1 fluorescence intensity shown in right panels (scale bar = 30 μm). (F) Immunofluorescence colocalization of IP3R (cyan) and VDAC1 (magenta) with corresponding quantitative analysis (scale bar = 30 μm). (G) Fluorescence microscopy images of lentivirus-infected hDPSCs (GFP signal indicates infection efficiency). (H) PERK silencing efficiency validated by Western blot and qRT-PCR. (I) Calcium flux analysis using Rhod-2 AM probe and representative fluorescence images. Quantification of calcium intensity (scale bar = 100 μm). (J) Transmission electron microscopy (TEM) images of MAMs structures (red asterisks indicate MAMs contacts; scale bar = 200 nm).

hDPSCs were stimulated with LPS at 0, 1, 10, and 20 μg/mL concentrations. qRT-PCR results showed that proinflammatory cytokines IL-1, IL-6, and TNF-α exhibited dose-dependent increases after 6 hours stimulation, peaking at 10 μg/mL and slightly declining at 20 μg/mL (Figure 3A). PERK expression levels were also dose-dependently upregulated, with significant protein expression increases (Figure 3B,C). MAMs-related genes IP3R, VDAC1, and GRP75 were significantly upregulated at 10 and 20 μg/mL LPS stimulation for 6 hours (Figure 3D).

Double immunofluorescence staining showed significantly increased PERK and VDAC1 expression at 10 and 20 μg/mL LPS stimulation for 6 hours, with maximum expression and colocalization observed at 10 μg/mL (Figure 3E). Similar trends were observed for IP3R and VDAC1, indicating quantitative changes in MAMs under inflammatory conditions (Figure 3F).

Lentiviral transfection of hDPSCs with ShPERK successfully reduced PERK expression, as confirmed by qRT-PCR and Western blot (Figure 3G,H). While MAMs-related genes showed no significant changes after LPS stimulation in ShPERK-transfected cells, Rhod-2 AM fluorescence staining indicated that silencing PERK prevented significant mitochondrial Ca^2+^ influx (Figure 3I). Transmission electron microscopy revealed that LPS stimulation (10 μg/mL, 6 hours) caused mitochondrial swelling, ER dilation, reduced MAMs distance, and increased MAMs numbers in the control group. These morphological changes were reversed in the ShPERK group, suggesting PERK’s role in MAMs formation under inflammation (Figure 3J).

### PERK impairs odontogenic differentiation via IP3R in inflammatory conditions

**Fig. 4:**
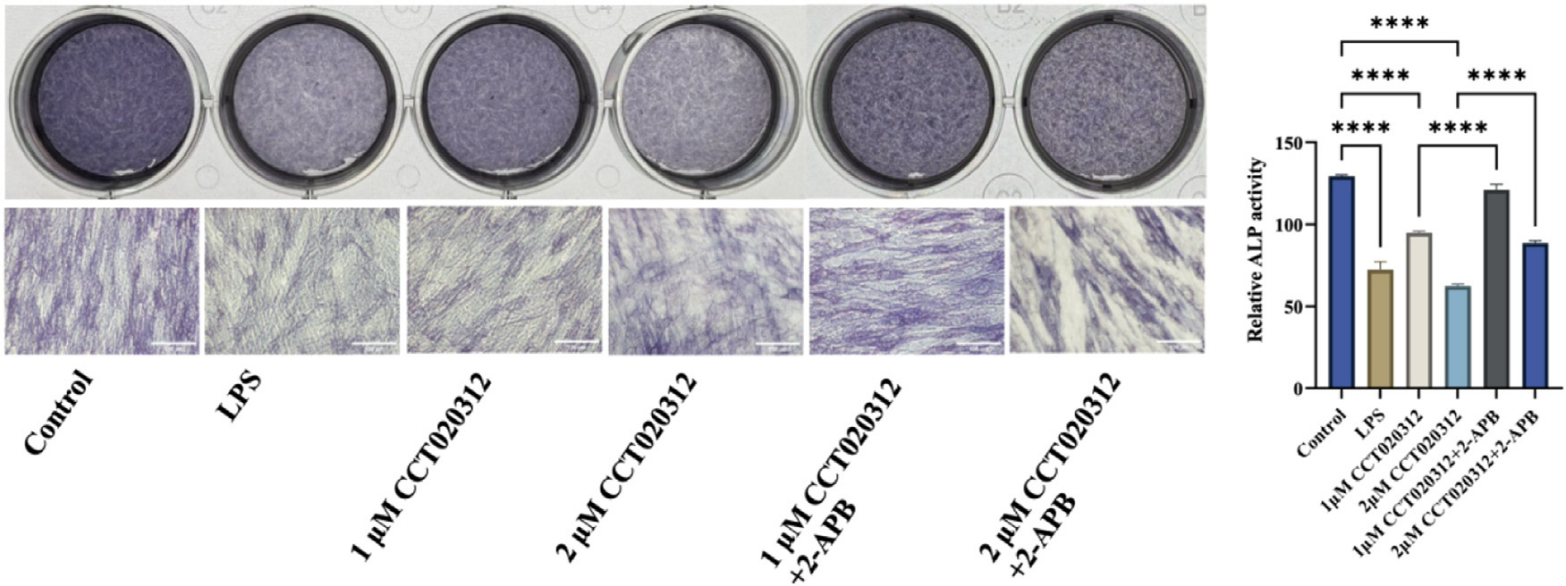
Alkaline phosphatase (ALP) staining results and ALP activity assay (scale bar = 100 μm).

LPS treatment significantly suppressed ALP activity in hDPSCs (Figure 4). Treatment with the PERK agonist CCT020312 further enhanced the decrease of ALP activity in a dose-dependent manner. While the inhibitory effect was reversed by coincubation with 50 μM IP3R inhibitor 2-APB, indicating the involvement of calcium signalling in PERK-mediated ALP regulation via IP3R.

## Discussion

Persistent dentin exposure establishes a pathological continuum wherein external stimuli progressively compromise the PDC, initiating intertwined inflammatory cascades, ERS, eventually leading to cell death and pulp necrosis. The functionality of hDPSCs plays a key role in facilitating effective and efficient tissue repair mechanisms within the dental pulp and dentin structures.^12^ Emerging single-cell transcriptomic evidence reveals that chronic inflammation induces metabolic reprogramming in hDPSCs, shifting them from tissue-reparative to proinflammatory phenotypes via ER-mitochondrial calcium cycling.^13^ Recent findings highlight PERK, a key stress-responsive kinase enriched at MAMs, as a critical molecular rheostat in this process.^5^ Our study demonstrated that inflammatory stimulation activated the PERK signalling pathway, which reprogrammed hDPSCs bioactivity by modulating ER-mitochondria coupling via MAMs to maintain intracellular homeostasis. This PERK-centric control coordinates hDPSCs fate decisions and provided a theoretical basis for the molecular mechanisms underlying pulp inflammatory challenge, extending the functional paradigm of UPR signalling into the realm of interorganellar communication in dental pulp biology.

Studies reported dual roles for PERK in osteogenesis, and no consensus has been reached. Knockout of PERK gene in mice murine models reduced type I collagen secretion and impaired osteoblast differentiation and bone development. Paradoxically, its activation in periodontitis models was associated with diminished osteogenic potential in periodontal ligament stem cells. This context-dependent duality may stem from PERK’s differential interactions with MAMs under varying pathological conditions. Our data shed light on this duality by implicating MAMs as a decisive platform. We observed pronounced ER-to-mitochondria Ca^2+^ transfer following LPS stimulation, concomitant with apoptosis induction and mineralization suppression. Crucially, silencing PERK attenuated mitochondrial Ca^2+^ overload, and pharmacological inhibition of the downstream IP3R calcium channel successfully restored mineralization capacity. This result corroborates findings that LPS enhances Ca^2+^ flow and provides direct evidence of PERK-mediated calcium dysregulation at MAMs in inflammatory osteogenic suppression.^14^ This was corroborated in vivo, where we observed spatiotemporal activation of PERK and its enhanced association with the MAMs component VDAC1 specifically at injury sites. This multilevel evidence supports a model wherein the inflamed pulp microenvironment heightens PERK-MAMs activity, leading to suppressed hDPSCs differentiation via IP3R-mediated Ca²⁺ dysregulation. The conclusion is further strengthened by our transcriptomic data, as KEGG analysis of the ShPERK group revealed a significant enrichment of calcium signalling pathway, and differential expression analysis showed that a majority of calcium signalling-related genes were markedly upregulated, including critical regulators like IP3R and CACNA1D. This finding provides strong, independent evidence that PERK is a master regulator of genes governing calcium homeostasis, underscoring its central role in the calcium flux dysregulation observed functionally.

Our study primarily utilized ALP activity as a key a sensitive early-phase readout for odontogenic differentiation. This approach was chosen to dissect the initial, decisive events whereby inflammatory signalling via the PERK-MAMs axis disrupts the commitment to differentiation. The rapid responsiveness of ALP to both LPS and pharmacological modulators made it an ideal parameter for our mechanistic study aimed at establishing causal links. Critically, the functional rescue of ALP activity upon IP3R inhibition directly links our proposed calcium dysregulation mechanism to a critical early step in the odontogenic program. Thus, the PERK-driven disruption of MAMs and calcium homeostasis, observed in vitro, provides a plausible mechanistic explanation for the suboptimal reparative dentin formation often seen in persistent inflammatory conditions in vivo. Future work will explicitly examine the impact of the PERK-MAMs-calcium axis on later differentiation stages, including to assess the expression of late markers such as dentin sialophosphoprotein (DSPP) and dentin matrix protein 1 (DMP1), along with terminal matrix mineralization via assays like Alizarin Red S staining. Furthermore, employing multiplex immunohistochemistry to spatially colocalize PERK activation with these markers in sequential injury models will provide direct in vivo correlation and validation.

In addition, the pathological role of the PERK-MAMs axis cannot be viewed in isolation. Recent work newly discovered that PERK activation could redirect calcium flux from cytosol to nuclear envelope via Sec61α translocation,^15^ potentially disrupting the calcium microdomains essential for dentin sialophosphoprotein (DSPP) expression.^16^ This potential mechanism is supported by our RNA-seq findings that TNF, IL-17, and NOD-like receptor signalling pathways significantly enriched under LPS stimulation, which provides a crucial upstream context for PERK activation. TNF-α signalling is a known potent inducer of ERS and has been shown to directly inhibit the proliferation, migration, and mineralization potential of hDPSCs.^17^ Similarly, IL-17, a key driver in pulpitis, can exacerbate inflammation and promote cell death via pathways intersecting with MAPK signalling.^18^^,^^19^ Notably, ERS itself has been shown to promote IL-17 expression, creating a potential feed-forward loop that sustains the inflammatory microenvironment. The NOD-like receptor pathway activates key signalling nodes like NF-κB and MAPK, further amplifying inflammatory cytokine production in hDPSCs.^20^ Furthermore, the significant enrichment of MAPK pathway genes upon PERK silencing aligns with emerging evidence that MAPK1 can disrupt MAMs integrity, thereby leading to substantial alterations in cell lipid metabolism.^21^ This connection is exemplified in related pathological contexts; for instance, inhibition of mitochondrial fission (a process regulated by MAPK) has been shown to ameliorate apical periodontitis by attenuating inflammasome-mediated inflammation.^22^ Critically, these inflammation-associated pathways are all known to intersect with and perturb calcium signalling and mitochondrial homeostasis. Their enrichment, therefore, outlines the broader inflammatory signalling network likely hijack the PERK-MAMs hub as a common downstream effector to dictate cell fate, placing it at a critical integrative node in hDPSCs’ response to injury. Moreover, the impact of PERK activation at MAMs likely extends beyond impairing differentiation. Recent evidence from intestinal inflammation models also observed increased MAMs numbers and PERK pathway activation, with pharmacological inhibition of PERK alleviating symptoms.^23^ Analogously, cellular stress in other dental tissues, such as serum starvation in periodontal ligament cells, can regulate cell fate through distinct organelle stress pathways like the ROS-AMPK/mTOR axis.^24^ This broader view suggests that in the inflammatory milieu, the PERK-MAMs axis orchestrates a coordinated pathological program affecting hDPSCs survival, redox balance, and regenerative potential, collectively contributing to pulp necrosis.

Regarding how PERK orchestrates MAMs function, our data point to a multilayered mechanism. The integrity of the MAMs complex, particularly proteins like VDAC1, is crucial for maintaining cellular homeostasis. Inflammation can disrupt VDAC1 function, leading to adverse consequences such as mitochondrial DNA release.^25^^,^^26^ VDAC1, which forms physical connections with IP3R and GRP75 to regulate Ca²⁺ and metabolite flux,^27^^,^^28^ represents a key functional component within the PERK-modulated nanodomain. Our data show that silencing PERK reversed LPS-induced mitochondrial swelling and ER expansion, confirming its role in MAMs stability. Specifically, PERK is required for the LPS-induced upregulation of IP3R mRNA, suggesting an indirect transcriptional or post-transcriptional role, potentially mediated by downstream effectors like ATF4. However, the rapid PERK-dependent remodelling of MAMs ultrastructure and calcium flux points to additional, likely post-translational, mechanisms influencing IP3R activity or complex assembly.^29^ Therefore, the precise mechanism by which PERK regulates IP3R remains a key open question. We propose two nonmutually exclusive hypotheses for future investigation: (1) Kinase-dependent regulation: As a serine/threonine kinase, PERK may directly phosphorylate IP3R or its regulatory proteins, thereby modulating its channel gating properties or sensitivity to inositol trisphosphate. (2) Scaffold-dependent complex assembly: Given its enrichment at MAMs and its critical role in maintaining ER-mitochondria contact integrity independent of its kinase activity,^11^ PERK may act as a scaffolding platform that facilitates the efficient formation or stability of the IP3R-GRP75-VDAC1 calcium nanodomain. Direct experimental evidence, such as coimmunoprecipitation to examine PERK-IP3R interaction, phosphoproteomic analysis, or fluorescence resonance energy transfer (FRET) assays to measure real-time complex dynamics under inflammatory conditions, will be crucial to distinguish between these mechanisms. Elucidating this aspect remains a primary goal of our ongoing research.

Collectively, our findings align with emerging ‘ER-mitochondrial synapse’ theory, where specialized membrane contact sites facilitate rapid, localized signalling. We propose that in the inflammatory microenvironment, PERK may function as both scaffold and signalling amplifier, modulating critical second messengers like calcium. This dual role makes it a central node in determining hDPSCs plasticity. A critical future question involves defining the threshold at which PERK activity shifts from a protective stress response to a driver of pathological MAMs hyperactivation. Future investigations may employ optogenetic calcium manipulators to spatially resolve PERK’s compartment-specific calcium regulation, or Cutting-edge organelle interactome mapping techniques (APEX2-based proximity labelling) could further elucidate PERK’s dynamic partnerships within MAM nanodomains during odontogenic differentiation.^30^^,^^31^ Beyond elucidating a fundamental cellular mechanism, our findings carry potential implications for regenerative endodontics. Clinically, conditions like symptomatic irreversible pulpitis are characterized by a dysregulated inflammatory microenvironment that hampers the innate regenerative capacity of the pulp. Our data suggest that overactivation of this axis may underpin the failure of adequate reparative dentin bridge formation in such scenarios. Therefore, strategically mitigating PERK signalling or stabilizing MAMs function locally – for instance, through the development of novel bioceramic or hydrogel-based pulp capping materials incorporating specific inhibitors – could represent a promising strategy to shift the balance from inflammatory degradation towards constructive repair. Future studies validating the upregulation of PERK and MAMs components in human inflamed pulp tissues, and testing the efficacy of targeted interventions in preclinical large-animal models, will be crucial steps towards translating this mechanistic knowledge into clinical application.

## Author contributions

Conceptualization: R.L.; Writing the original draft: Y.L.; Review and editing: Y.L., Y.W., Z.L., Y.J., R.L.; Figure conception and preparing: Y.J.; Manuscript revision: Y.L., Y.W.

## Funding

This study was funded by the Shaanxi Natural Science Foundation (grant number 2022JZ-56).

## Conflict of interest

The authors declare that they have no known competing financial interests or personal relationships that could have appeared to influence the work reported in this article.

## Appendix Supplementary materials

Image, application 1
