# Supplementary material for: Protein Kinase R-like Endoplasmic Reticulum Kinase-Mediated ER-Mitochondria Coupling Regulates Odontogenic Differentiation of Human Dental Pulp Stem Cells Under Inflammatory Stimuli
Source: Int Dent J. 2026 Mar 5;76(3):109440. doi: 10.1016/j.identj.2026.109440 (PMC12970396; doi:10.1016/j.identj.2026.109440)
Supplement: Supplementary file 1 [file mmc1.docx]

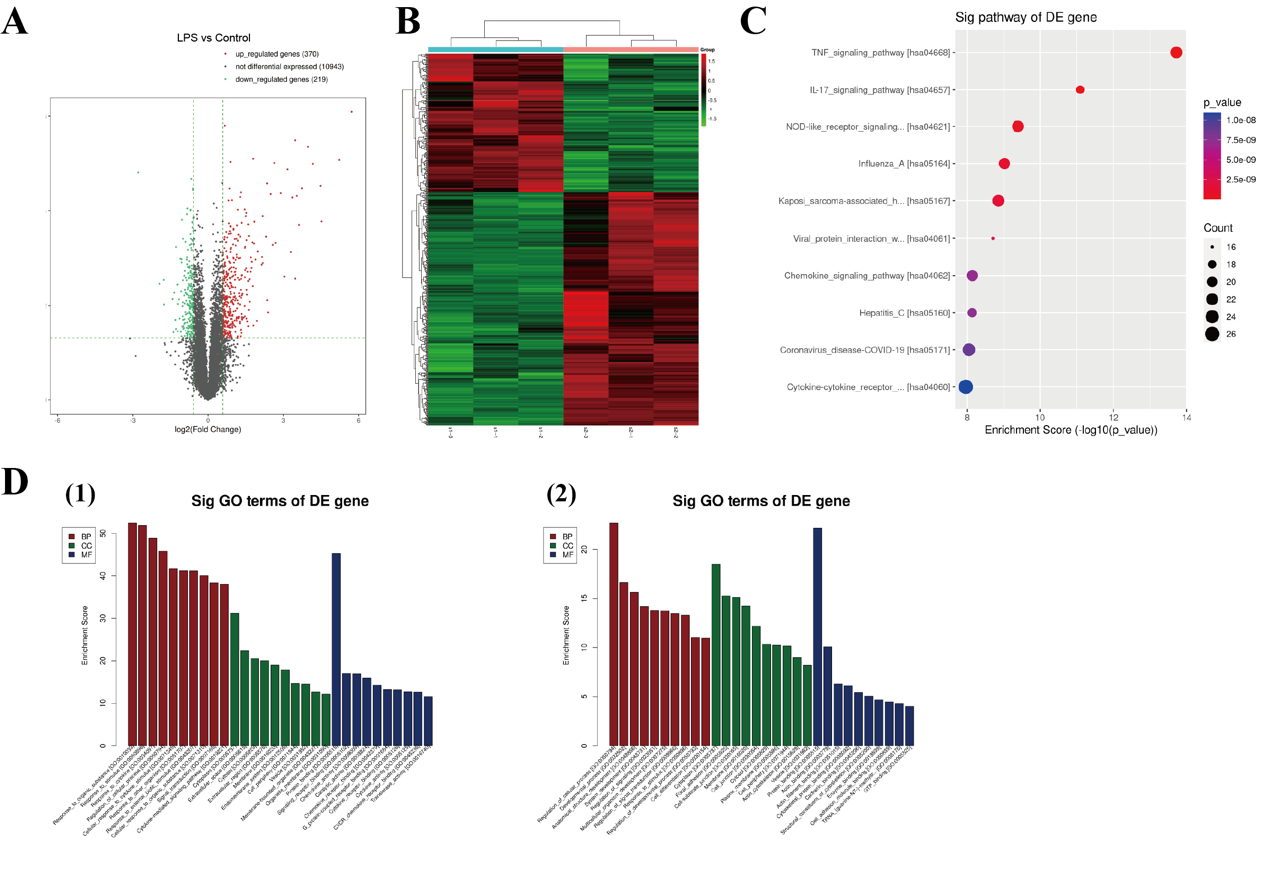


**Supplementary Fig S1** Transcriptional profile of hDPSCs under LPS-induced inflammatory stimulation. (A) Volcano plot of DEGs between LPS and Control groups (red: 370 upregulated genes; green: 219 downregulated genes; |log2FoldChange| > 1 and P-value < 0.05). (B) Hierarchical clustering analysis of DEGs between LPS and Control groups. (C) KEGG enrichment bubble plot of DEGs in LPS vs Control groups. (D) GO enrichment analysis of DEGs in LPS vs Control groups:(D1) Top 10 GO terms significantly enriched among upregulated DEGs. (D2) Top 10 GO terms significantly enriched among downregulated DEGs.

Supplemental Table 1. NanoDrop-1000 RNA Quality Assessment

| sample name | OD260/280 | OD260/230 | concentration  (ng/μl) | Volume  (μl) | quality  (ng) | QC |
| --- | --- | --- | --- | --- | --- | --- |
| s1-1 | 1.94 | 2.02 | 358.17 | 100 | 35817 | passed |
| s1-2 | 1.97 | 1.98 | 293.79 | 100 | 29379 | passed |
| s1-3 | 1.99 | 2.18 | 716.86 | 100 | 71686 | passed |
| s2-1 | 1.93 | 2.22 | 469.11 | 100 | 46911 | passed |
| s2-2 | 1.97 | 1.9 | 389.89 | 100 | 38989 | passed |
| s2-3 | 1.96 | 1.91 | 342.97 | 100 | 34297 | passed |
| ShPERK-1 | 2.17 | 1.98 | 47.5 | 100 | 4750 | passed |
| ShPERK-2 | 2.13 | 1.92 | 81.9 | 100 | 8190 | passed |
| ShPERK-3 | 2.16 | 1.9 | 81 | 100 | 8100 | passed |

**Note:** The OD260/OD280 ratio for pure RNA should be close to 2.0 (acceptable range: 1.8-2.1), and the OD260/OD230 ratio should be >1.8.

Supplemental Table 2. NanoDrop-1000 RNA Quality Assessment

| sample name | Reads | Q30（%） |
| --- | --- | --- |
| s1-1 | 47134868 | 94.48% |
| s1-2 | 37361146 | 94.21% |
| s1-3 | 35196862 | 93.22% |
| s2-1 | 36756764 | 94.46% |
| S2-2 | 34081594 | 94.50% |
| S2-3 | 47317436 | 94.33% |
| ShPERK-1 | 34549342 | 97.17% |
| ShPERK-2 | 38529484 | 97.34% |
| ShPERK-3 | 36165858 | 97.17% |
